# Risky Trade: Infectious Disease in the Era of Global Trade

**DOI:** 10.3201/eid1410.080835

**Published:** 2008-10

**Authors:** Andrew Price-Smith

**Affiliations:** Colorado College, Colorado Springs, Colorado, USA

The linkages between facets of globalization and the emergence and recrudescence of infectious disease are a topic of increasing concern for providers of public health, international business concerns, economists, and political elites. Such interdisciplinary inquiry is rare, and sorely needed, at the dawn of the 21st century. Ann Marie Kimball’s book is a welcome addition to those few inquiries that cross the disciplines of epidemiology, economics, and political science. Jargon free, and well written, the book is an excellent analysis of the consequences of globalization upon public health and the consequences of disease for international trade and economic productivity.

The term globalization is frequently used in modern discourse but often poorly defined. Typically, it refers to the movement of financial capital and trade goods. However, a nuanced understanding of globalization accounts for negative externalities such as human-induced environmental change and the emergence and recrudescence of infectious diseases. Kimball addresses these linkages in deft fashion and notes the often negative consequences of the complex interactions between the worlds of trade, ecology, public health, and politics. Despite the great need for interdisciplinary approaches to deal with concatenating global problems, barriers between scientific disciplines, a “silo mentality,” persist and undercut our capacity to respond to emerging threats.

Kimball argues that globalization and its associated processes (crowding, poor sanitation, travel and trade, intensive food production practices, and ecologic change) all increase the threat of pathogen emergence. Her book explains the mechanisms by which ecologic change drives processes of pathogenic emergence, facilitates zoonotic transfers, induces mutation, and permits the globalization of antimicrobial drug resistance. Kimball also provides an illuminating analysis of the mechanics of microbial interdependence between the industrialized and nonindustrialized worlds. She argues that globalization has directly contributed to the emergence of pathogens such as the severe acute respiratory syndrome (SARS) coronavirus and is increasing the probability of pandemic influenza. In sum, she argues correctly, globalization is creating a new ecology of disease.

In the domain of trade and economics, Kimball provides an illuminating discussion of the global blood trade and its pivotal role in the emergence of the HIV/AIDS pandemic. Following that discussion, she provides a detailed analysis of the negative economic impact of infectious disease (e.g., SARS, influenza, cholera, bubonic plague) upon trade and economic productivity and notes the economic damage resulting from trade embargoes imposed upon countries that exhibit epidemic infections. Frequently, such embargoes are the result of uncertainty-induced fear and lack any empirical basis. Kimball also provides a useful overview and analysis of the “compulsory licensing” provisions, and the protocols for Trade-Related Aspects of Intellectual Property Rights of the World Trade Organization (WTO). In the event of a public health emergency, such protocols enable nations to develop lifesaving medicines that infringe upon the patents developed by others. Furthermore, she argues that the WTO does not effectively represent the interests of the nonindustrialized countries, particularly when the interests of international business conflict with the well-being of the indigent.

Kimball also does an excellent job of critiquing domestic and international forms of public health governance. She recognizes, accurately, that the state does indeed have a central role to play in pathogen surveillance, the judicious use of quarantine, and the provision of public goods such as healthcare. She discusses the interaction (and occasional tensions) between sovereign states and international organizations (e.g, the World Health Organization and WTO) and notes the limitations of international health regimes, such as the International Health Regulations, even in their recently revised form. Kimball discusses the perils induced by low levels of surveillance and containment capacity in the nonindustrialized countries, noting that serious outbreaks of contagion frequently overwhelm local health infrastructures and health providers. Consequently, she stresses the need to bolster global pathogen surveillance, diagnostic, and response networks. Unfortunately, as Kimball duly notes, public health remains rather marginalized in the conduct of international politics and in the study of international relations as well.

Finally, the author provides an excellent critique of health governance at the domestic level within the United States. She begins with a discussion of protocols for domestic biodefense and briefly analyzes the utility of exercises such as Global Mercury, Dark Winter, and Top Officials (TOPOFF). Kimball questions the efficacy of the National Pharmaceutical Stockpile and notes the vulnerability of the US food supply. Further, she notes the lack of autonomy (and often capacity) of those divisions of the US bureaucracy tasked with the protection of public health, particularly the US Department of Agriculture. Kimball astutely warns of the perils of the self-congratulatory “happy talk” so prevalent in international organizations, national governments, and nongovernmental organizations, as it leads to overestimation of response capabilities. Finally, Kimball warns that the lack of universal health insurance in the United States actually increases societal vulnerability to contagion.

In sum, this is a very good book, well-suited to public health practitioners and medical personnel, and senior undergraduates. And, frankly, it should be read by those in the realms of business and politics as well.

